# Big Data Precision Marketing Approach under IoT Cloud Platform Information Mining

**DOI:** 10.1155/2022/4828108

**Published:** 2022-01-12

**Authors:** Wang Li

**Affiliations:** Business School, Xijing University, Xi'an, Shaanxi 710123, China

## Abstract

In this article, an in-depth study and analysis of the precision marketing approach are carried out by building an IoT cloud platform and then using the technology of big data information mining. The cloud platform uses the MySQL database combined with the MongoDB database to store the cloud platform data to ensure the correct storage of data as well as to improve the access speed of data. The storage method of IoT temporal data is optimized, and the way of storing data in time slots is used to improve the efficiency of reading large amounts of data. For the scalability of the IoT data storage system, a MongoDB database clustering scheme is designed to ensure the scalability of data storage and disaster recovery capability. The relevant theories of big data marketing are reviewed and analyzed; secondly, based on the relevant theories, combined with the author's work experience and relevant information, a comprehensive analysis and research on the current situation of big data marketing are conducted, focusing on its macro-, micro-, and industry environment. The service model combines the types of user needs, encapsulates the resources obtained by the alliance through data mining for service products, and publishes and delivers them in the form of data products. From the perspective of the development of the telecommunications industry, in terms of technology, the telecommunications industry has seen the development trend of mobile replacing fixed networks and triple play. The development of emerging technologies represented by the Internet of Things and cloud computing has also led to technological changes in the telecommunications industry. Operators are facing new development opportunities and challenges. It also divides the service mode into self-service and consulting service mode according to the different degrees of users' cognition and understanding of the service, as well as proposes standardized data mining service guarantee from two aspects: after-sales service and operation supervision. A customized data mining service is a kind of data mining service for users' personalized needs. And the intelligent data mining service guarantee is proposed from two aspects of multicase experience integration and group intelligence. In the empirical research part, the big data alliance in Big Data Industry Alliance, which provides data mining service as the main business, is selected as the research object, and the data mining service model of the big data alliance proposed in this article is applied to the actual alliance to verify the scientific and rationality of the data mining service model and improve the data mining service model management system.

## 1. Introduction 

Industrial integration refers to the dynamic development process in which different industries or different sectors of the same industry penetrate and cross each other and merge into a whole, gradually developing into a new industry. Industrial integration is not only studied as a development trend, currently, but industrial integration has also developed into a realistic choice for the development of the communications industry [1]. From the perspective of the development of the telecommunications industry, in terms of technology, there is a trend of mobile substitution for fixed networks and triple-play convergence in the telecommunications industry, and the development of emerging technologies represented by the Internet of Things and cloud computing has also led to technological changes in the telecommunications industry, making telecommunications operators face new development opportunities and challenges. In terms of business, full-service operations have been used as the main mode of operation in the telecom industry around the world [2]. Big data trading platforms led by data production or service enterprises have become the main medium for data trading activities, and these enterprises have actively explored in specific fields and industries and formulated data trading rules, norms, and targets for their respective platforms, but there are generally unclear boundaries in functional positioning, forming multiple-segmented trading markets, making it difficult to achieve scale and industrial development, leading to low mobility of data resources [3]. This requires enterprises in different fields to integrate or cooperate as well. These companies have made active explorations in specific fields and industries, and formulated data transaction rules, specifications, and goals for their respective platforms. However, there are generally unclear boundaries in functional positioning, forming multiple-segmented trading markets, and it is difficult to achieve scale. The development of globalization and industrialization has led to low data resource mobility. Although enterprises with strong capital can supplement the data by acquiring small enterprises, one enterprise cannot achieve the comprehensive data required by market demand anyway, not to mention that acquisition activities are also unattainable for many SMEs. Therefore, many geographically dispersed data resource-based enterprises, technology-owned enterprises, service-application enterprises, network business enterprises, infrastructure enterprises, etc. The big data industry chain relies on advanced Internet and mobile Internet and other communication methods to provide data services as the mainline, by bridging the data resource gap of alliance members, in data transactions and big data industry operations, to complement each other with resource advantages [4]. By bridging the data resource gaps of the alliance members, the alliance aims at complementing resource advantages, innovating business service models, and enhancing their competitive advantages in data trading and big data industry operation, and finally forms a new cross-enterprise organization form of benefit sharing and risk sharing—big data alliance.

Although the IoT public cloud platform is developing fast and provides comprehensive functions, public cloud can support more user access and reduce cloud platform load, the public cloud access is mainly HTTP, HTTP is suitable for some devices with low sensitivity to real time, and the devices with high sensitivity to real-time are not suitable for accessing the public cloud, such as logistics monitoring in industrial production, which requires real-time monitoring logistics information flow, and when you need to control or send orders to a certain aspect of the logistics chain, you need very good real time to ensure that the orders are delivered accurately and on time. And in some enterprises, enterprises have high-security requirements for data in production, such as security monitoring, and some design enterprise originality and commercial secrets of data cannot be stored in the public cloud platform, such as the collection of enterprise equipment data, and enterprises need their servers to save equipment data [5]. What enterprises need is a platform that can meet the production needs of enterprises, especially for domestic small and microenterprises, enterprises focus on different directions, the product data in the enterprise are also different, the demand for the platform is also different, so the public cloud platform is not suitable for diverse needs. For different data needs of different enterprises, scalability should be built to meet the needs of enterprise data platform, so a private platform for different enterprises with different data requirements should be built, a scalable platform should be built to meet the data requirements of enterprises, so building a private platform for enterprise IoT is more capable of promoting the combination of IoT technology and actual enterprise production [6].

The construction of the data aggregation service model of the original-level data aggregation service, the feature-level data aggregation service, and the decision-level data aggregation service of the big data alliance enables the members of the Alliance to break through their development bottlenecks and form a value-added industrial chain with the sharing of data resources as the cooperation link and promotes the virtualized cross-regional operation of the Big Data Alliance; on the other hand, with the data warehouse as the centre, relying on the Big Data Alliance data aggregation service model, it can provide convenient and real-time quality data services for the majority of users. Therefore, the data aggregation service model of building high-quality original-level data aggregation service, feature-level data aggregation service, and decision-making-level data aggregation service for big data alliance can rapidly accelerate the efficiency of using and sharing data resources of big data alliance and, finally, realize the value-added of data resources of big data alliance. Taking data services as the main line, by making up for the data resource gap of alliance members, in the operation of data transactions and big data industry, with the goal of complementing resource advantages, innovating business service models, and enhancing its own competitive advantages, it has finally formed benefit sharing and risk sharing a new cross-enterprise organization form.

## 2. Related Works

Nowadays, the competition between the major giants in the field of e-commerce is becoming more and more fierce, and in the way of marketing coordination, the effective self-operated coordination and precision marketing strategy has been more widely used and achieved certain results. As an emerging industry and tool, with the rise of e-commerce, precision marketing is also a new thing in the advertising industry, and the research results are not supported by specific systems [7]. But it is undeniable that precision marketing in today's new network business model is a more reduced cost, can bring accurate traffic means of communication, can be for small- and medium-sized enterprises in the industry in the case of tall buildings for more benefits, and have their foothold [8]. For large enterprises, accurate customer can greatly save publicity costs, and operating costs can make the enterprise to the customer's service more personalized. Future-oriented precision marketing is coming soon due to the role of capturing people's needs in the mainstay of the advertising industry [9]. With the rapid spread of information, the concept of precision marketing has become familiar and is gradually developing into a new marketing concept. For this reason, based on the background of the rapid development of e-commerce, the connotation of personalized precision marketing and its characteristics will be targeted research, and its marketing strategy will be analyzed and discussed [10]. Personalized precision marketing has several characteristics and advantages, and the main strategies for e-commerce enterprises to carry out personalized precision marketing are to improve the level of personalized services, accelerate the process of enterprise informatization, and develop the precise positioning of enterprises [11].

The target market is the market demand that the enterprise must meet in the process of marketing, that is, the object of the enterprise's service [12]. No enterprise or product can meet the overall needs of all consumer markets, so it can only choose a certain range of target markets for the enterprise as well, and market positioning is a certain market position that the enterprise specifies to highlight its products according to the consumer demand level. After selecting the target market, due to market competition and a wide range of information, to gain market advantage, the enterprise must form a product with different characteristics from its competitors after understanding their characteristics to form a strong impression in the minds of consumers. The purpose of market positioning is to gain a competitive advantage and attract more potential consumers. This theory is very applicable to operator big data marketing [13]. This is because the marketing of operators' big data is not only to sell the operators' data to the target customers after “desensitization,” but also to bundle the operators' big data with the customers' interest chain to achieve a solution where everyone benefits, and as a communication service provider, good service is crucial to the development of big data business [14].

Big data marketing generally includes four processes such as data collection, data mining, and analysis, predictive analysis, and data result in feedback. Compared with traditional marketing methods, big data marketing has advantages such as high timeliness, individual customization, high-cost effectiveness, and relevance promotion. The advent of the era of big data has brought great changes to social production and life. As a representative innovative technology, the mobile Internet has brought massive user behavior data, providing unprecedented space and potential for industries and enterprises to obtain deeper and more comprehensive insights, which makes big data marketing break through the boundaries of Internet companies, so that all walks of life can have the opportunity to have an in-depth understanding of their market environment, service targets, customer groups, or even competitors. The economic environment is an important factor in realizing demand. The operation and development of the economic environment will directly or indirectly affect the market decisions of enterprises, and it is mainly expressed in marketing as the level of economic development of a country or region and the purchasing power of consumers, which determines the size of the enterprise market.

## 3. IoT Cloud Platform Big Data Information Mining Precision Marketing Analysis

### 3.1. IoT Cloud Big Data Platform Construction

The overall architecture of the enterprise-oriented IoT cloud platform system needs to be designed from the perspective of data and enterprise users. For data, the flow between the data from the collection device to the storage system needs to be considered, and for enterprise users, the cloud platform only needs to provide users with the services they need, and the internal implementation of the cloud platform and the underlying data collection is transparent to the users [15]. Therefore, the architecture needs to meet the function of each subsystem, determine the responsibilities between each subsystem and the interface definition of other subsystems, and conform to the principle of high cohesion and low coupling defined by software engineering, and the overall architecture is easy to maintain and expand later. For example, security monitoring, and some design companies' original and commercial secret data cannot be stored on public cloud platforms. For example, corporate equipment collects data, and companies need their own servers to store equipment data. The system provides relevant services in the form of interfaces and only needs to explain the interface-related use issues and use methods, and the internal implementation logic is transparent to the user, and the user only needs to operate in the local client to complete the relevant tasks.

**Figure 1:**
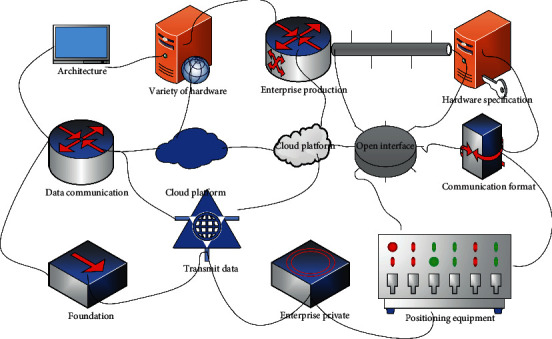
Structure of communication services.

Data communication is the foundation of the enterprise private cloud platform and the starting point of the cloud platform architecture. In enterprise production, due to the wide variety of hardware, each hardware specification is different, communication format and device access are different, such as vehicle positioning equipment can directly connect to the open interface of the cloud platform to transmit data, but some small embedded devices such as GPS equipment cannot directly use the HTTP protocol to transmit data, so it needs embedded gateway as middleware to transmit data. Due to the different kinds of equipment measurement data and the different functions of each device, the data reporting period of some devices is very long; for example, the level meter in safety monitoring only needs to report one data a day, which does not require high real time, but, for example, in the MES workshop intelligent manufacturing system, it needs to monitor the state of the material transportation process in real time, so it needs to upload data every minute or even every 30 S or shorter period. This kind of equipment has high requirements for real-time data and for real-time commands issued by the cloud platform, as shown in Figure 1.

For the different data requirements of different enterprises, a scalable platform that meets the data needs of the enterprise should be built. Therefore, building a private platform for enterprise Internet of Things can further promote the combination of Internet of Things technology and actual enterprise production. The enterprise private IoT cloud platform needs to be able to be compatible with the connection of different types of devices with different real-time requirements. The whole communication system has three layers: the bottom device and embedded gateway, the intermediate device access interface layer, and the cloud platform data processing layer. For the device layer and the interface layer, due to the different real-time requirements of the devices, the cloud platform provides two types of device access: HTTP protocol access, and TCP protocol access. Since the cloud platform must support many devices, to improve the device access capability of the cloud platform, the combination of HTTP and TCP is used to complete device access. First, for devices with high real-time requirements, the cloud platform provides a real-time device access interface, and devices can directly connect to the cloud platform through the TCP to directly transmit data, and the cloud platform can actively send commands to the devices to ensure the real-time data upload and command delivery [16]. Netty is a highly available multiplexed network framework, and the underlying poll implementation can support highly concurrent connections and data exchange to improve the efficiency and speed of the system.

The data storage subsystem is built by combining the relational database MySQL with the nonrelational data available MongoDB cluster, which ensures that all data can be efficiently stored and queried through a unified data access layer to access different formats and types of data. The system uses the MySQL database for the storage of structured data, such as user data, application data, device data, system log data, and device configuration, which is used to realize the structured storage and fast access of correlated data. As the cloud platform accesses different types of devices, the data format and data dimensions collected by the devices are different, and it is difficult to store unstructured data with a relational database, the system selects MongoDB for unstructured IoT data with the simple data model, strong flexibility, huge data volume, and high requirements for data access. It can quickly accelerate the use efficiency and sharing efficiency of data resources of the big data alliance and ultimately realize the value-added of the data resources of the big data alliance.

**Figure 2:**
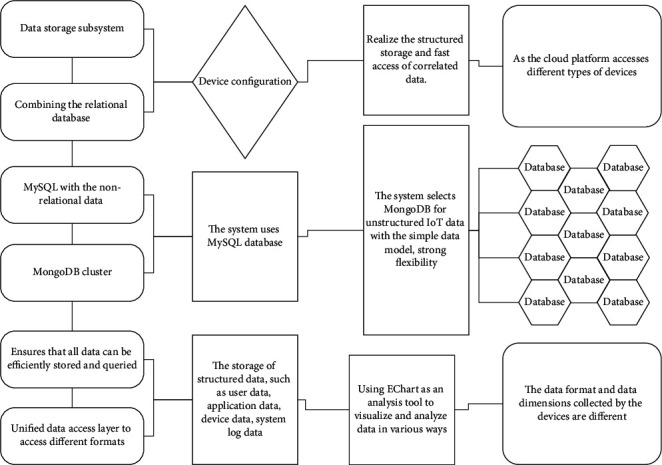
IoT cloud platform cluster solution diagram.

The cloud platform needs to provide enterprises with a range of data-based services to visualize and analyze data, using Echarte as an analysis tool to visualize and analyze data in various ways. Use Baidu Map API to provide location services for users with location needs. The platform open development interface, the caller can cognize the function of the interface role, convenient to call the development of personalized applications, as shown in Figure 2.

The IoT cloud platform consists of a communication server cluster, management server cluster, MongoDB cluster, and MySQL cluster; the communication server cluster is responsible for accessing the underlying devices, processing device uploads, processing user commands, pushing real-time data, and providing application development interfaces. The MongoDB database cluster is responsible for accessing the uploaded data of devices, and the MySQL database cluster is responsible for storing relational data of users and devices in the cloud platform.

After the device establishes a connection with the gateway, the acquisition module of the device is in the dormant state, the device does not upload data and sends heartbeat information to the gateway every time, the gateway receives the heartbeat information and parses the heartbeat, and uploads it to the cloud platform after the cloud platform receives the heartbeat information, it does not query the issued command of the device in the pending issued command table, the cloud platform replies to the gateway with the heartbeat confirmation, and the gateway receives the heartbeat confirmation. After receiving the heartbeat confirmation, the gateway sends an acknowledgment frame to the device, and the device is disconnected from the gateway. When the device acquisition module starts to collect data, the device sends the original acquisition data to the gateway, the gateway parses and re-encapsulates the acquisition data and sends it to the cloud platform, after the cloud platform receives the acquisition data and does not query the down command of the device in the pending command table, the cloud platform returns the data confirmation to the gateway, the gateway sends the data confirmation frame to the device after receiving the data confirmation, and the device is disconnected from the gateway.

When the acquisition module is collecting data, the cloud platform sends commands to the device. The device sends raw data to the gateway at regular intervals, the gateway forwards it to the cloud platform, the cloud platform receives the acquisition data and brings back the command sent by the management system to the gateway, the gateway receives the command and encapsulates the command into a command frame and sends it to the device, the device confirms the command after receiving it and sends the command confirmation frame to close the connection, the gateway sends the command confirmation message to the cloud platform, and the cloud platform transfers the command from the to-be-sent. The cloud platform transfers the commands from the command table to the already sent command table after receiving the command confirmation information.

The concept of putting the customer first has swept the world, and we are increasingly looking at what the customer thinks. Precision marketing is essentially the optimization and upgrading of segmented communication, and this model is beginning to be favoured by business owners and advertising agencies [17]. The development of precision marketing has gone through three cycles: the first stage is defined as the targeting of regional advertising messages; the second stage is the targeting of personalized customer preferences; and the third stage is the development of mobile network technology and the establishment of a personal database of Internet users, which collects and establishes a database and analyses the personalized needs of users, and then uses programmatic means to bid for advertising space in real-time, to present more precise advertising services to the public. The third stage is the development of mobile web technology and the establishment of a personalized database of web users, collecting and building the database and analyzing their personalized needs.

### 3.2. Information Mining Precision Marketing Design

The tag system refers to the formation of a user tag system based on basic user data through aggregation and inductive analysis, which is not directly operated externally but only used as part of the data source for precision marketing products and other products such as the capability open platform. Data capability open platform is based on the demand of partners for data and platform resources, for partners to share Unicom data resources and infrastructure resources in a multitenant secure isolated way by opening the sample data with user identification information removed in the Unicom environment. Conduct in-depth discussions and research, and formulate corresponding marketing improvement countermeasures, which have a certain enlightening effect on the company's optimization of marketing strategies. Digital marketing is relying on massive data and big data processing capability, and under the premise of ensuring data privacy and security, optimizing the placement strategy and channels for customers in various industries through in-depth mining and analysis of big data, providing accurate marketing reach means to achieve the purpose of reducing marketing costs, and improving marketing effectiveness. Smart Footprint is to provide big data analysis services of user groups such as population density, cross-domain migration, and travel analysis for urban planning, traffic planning, and outlet location by pinpointing the number of populations within a certain granularity.

Internet data are limited by its own data genes, which are manifested in the following four aspects: first, the data of Internet companies are fragmented, e-commerce only has e-commerce sales data, no Baidu search data, and the data exists locally; second, due to company strategy and data security requirements, few Internet companies are willing to open their data to partners or competitors. Even if there is openness, more are only at the business model level and application level, and the data level is still relatively closed; third, Internet data integration is difficult, and the registered personal account is also short-term, and the account information is unstable; and fourth, the business field of Internet companies directly determines the content of data, and the scope of data is not comprehensive. Data flow requires multipart cooperation, so the architecture needs to clarify the functions between each subsystem, determine the responsibilities between each subsystem, and define the interface for other subsystems.

(1) $$\begin{array}{c} u_{A}=qk_{0}\left(\beta_{A}-\delta_{A}\xi_{BA}-\delta \beta \alpha_{AB}+q_{0}k_{0}\zeta_{AB}\right),\\ u_{A}^{\prime }=qk_{0}\left(\beta_{A}+\delta_{A}\xi_{BA}-\delta \beta \alpha_{AB}-q_{0}k_{0}\zeta_{AB}+w\right). \end{array}$$

**Figure 3:**
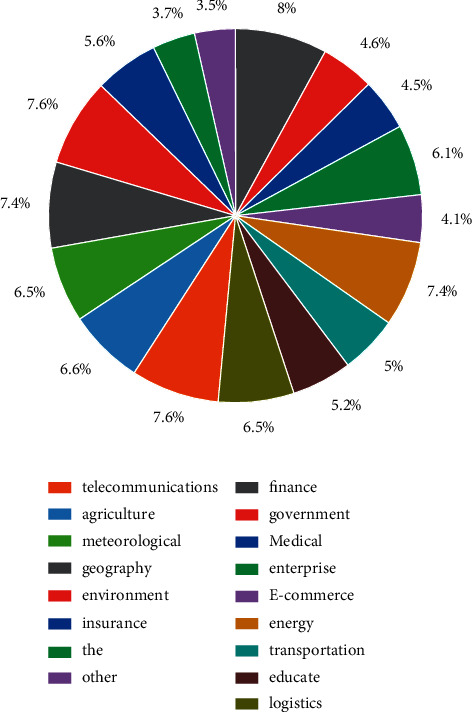
Market share of big data applications by industry.

Through the division of labour and cooperation, member companies in the big data alliance jointly develop the technologies required in the field of big data processing and provide data service solutions for users, and data resource sharing runs through the entire data service-application development process of the alliance [18]. Combined with the previous analysis process of the equilibrium state of data resource sharing in a big data alliance, the growth process of data resource stock of each member under the influence of the data resource sharing coefficient is shown in Figure 3. The results show that the data resource sharing coefficient shows a positive feedback relationship with the equilibrium state of data resource sharing, which directly affects the equilibrium point values under different sharing relationship patterns. By adjusting and coordinating the coalition data resource sharing coefficients, the data resource stock growth will show different equilibrium states.

Data aggregation refers to the use of scientific aggregation methods to orderly connect the scattered and isolated data resources scattered among different members of the big data alliance in a certain way, and implement the structural system rebuilding, so that data resources from different sources, at different levels, and with different contents can be integrated comprehensively, thus forming a valuable and complete system. Data correlation refers to the value transformation process of data to information (knowledge) by extracting information or extracting some knowledge from data resources by organizing and extracting features in data resources based on correlation analysis, and achieving some interesting discoveries. Data fusion refers to the process of conversion, integration, and merging in information or knowledge by using advanced big data processing technology to further concentrate and refine to form new information and new knowledge, to construct effective information resources and knowledge resources for users' domain problems-solving and release the deep potential value of big data:

(2) $$\begin{array}{c} {\text{POS}}_{p}{\left(Q\right)}_{\left(x\right)}=\cup PX\left(x^{2}\right),\\ \prod_{B}^{A}A\left(I_{1},I_{2},\ldots ,I_{m}\right)=\underset{a_{j}\in B}{\cap }Ajl_{j}^{2}. \end{array}$$

From the perspective of value cocreation, data aggregation is the data aggregation at the original level of the big data alliance, the data value breeding stage. In the very early days, data were considered valuable, but data that could breed great business value were not data in the traditional sense but should be referred to as data elements or data resources. This is because while data contain a great value, it needs to be further developed and utilized before the value of the data can be gradually released and amplified, which is essentially like elements and resources. In the operation of a big data alliance, whether the data resources scattered in different channels correctly and effectively brought together in the alliance is the key to the aggregation of data at the original level, which will directly affect the efficiency of the alliance operation and the final service results. It is necessary to consider the overall performance loss caused by the mutual calls between subsystems and the flow of data between the various subsystems, as well as the complexity of the cloud platform in the development process:

(3) $$\begin{array}{c} M_{B}=\left\{D_{k}^{2}|D_{B}^{2}\right\},\\ y\left(x\right)=\mathrm{sgn}\sum_{k=1}^{N}a_{k}\varphi \left(x,x_{k}^{2}\right). \end{array}$$

In general, core enterprises in big data alliances tend to be more willing to proactively share data resources to maintain their core position in the network, and noncore enterprises mainly play the role of supporting support. Data sharing will be more likely to occur when a mutually beneficial symbiotic relationship is formed among the member enterprises of the alliance. In particular, the more the values of different types of member enterprises in the alliance converge, the easier it will be to understand and trust each other in cooperation, which is conducive to forming a consensus and sense of identity for data sharing:

(4) $$\begin{array}{c} F=\arg\min\left(M_{K\times H}g_{H}^{2}\right). \end{array}$$

**Figure 4:**
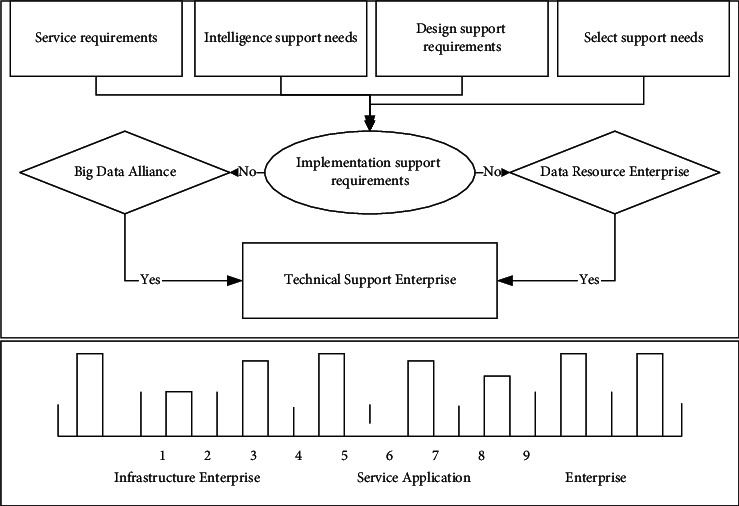
Schematic diagram of the formation of a decision-level data aggregation service offering.

A service tagging system is a tag library built around multiple entity objects and connections between entities using the method of tagging description, which is an important foundation for building portraits, graphs, and networks [19]. The big data alliance establishes a rich tag library through a combination of manual and machine tagging methods while monitoring the use and effects of various types of tags, counting popular and selected tags in real time, replacing unreasonable tags in the library, and continuously adding new tags according to the needs of the business to further promote the improvement of the big data alliance data tagging system and form a dynamic collection of data interpenetration-related service tags. At the same time, if users have many instances of data resources, they can support the classification management of instance resources on the platform by binding tags to the instance resources, so that users can customize the new tags, as shown in Figure 4. It includes the underlying device and embedded gateway, the access interface layer of the middle device, and the data processing layer of the cloud platform.

In the process of feature-level data aggregation services, the complex data structure at the bottom of the alliance is separated by the tag model view layer to achieve the flexible configuration of data resources, which can greatly enhance the operational experience of member enterprises in specific situations. Specifically, we try to flexibly build the required business modules on top of the tags, use the tags as tools to realize unified business computing operations on data, and support on-demand tag selection according to the needs of business scenarios. In the actual operation process, under the premise that the big data alliance needs to integrate the data resources of multiple members, this dynamic tag extraction approach has very good scalability [20]. In the whole service panorama modelling process, due to the limitations and inadequacies of a single method, the cross-fertilization of a rich system of multidisciplinary fusion methods is needed to support the flexible extraction of features from these massive data resources and to realize the reasonable allocation and scheduling of service resources in the service panorama modelling process to quickly respond to the personalized needs of users. big data alliance provides the function of sharing the same label attempt between multiple modules so that the results of data interpenetration correlation analysis can be further combined with algorithm module, tool module, and method module, and the objects screened by the analysis can be combined with other application systems, such as combining multiple application scenarios such as operation and maintenance management of intelligent transportation system, customer relationship management in the marketing field, and equipment supervision of enterprise production and operation. Help users to solve the core problems in specific field contexts, and improve their data service satisfaction.

## 4. Results and Analysis

### 4.1. IoT Cloud Big Data Platform Performance

**Figure 5:**
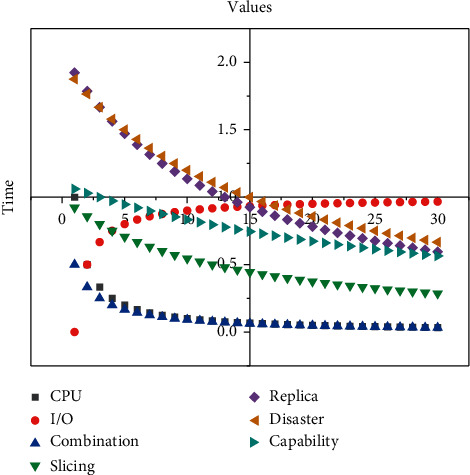
Real-time analyses of data from the cloud platform.

The shard mode is a way to slice the data horizontally, it includes a routing server mango, a configuration server mongo, and a slice server shard the data are stored by the slice server, the routing server is the entrance of the whole database cluster, and all read and write requests have to go through the routing server to distribute the requests to the corresponding. All read and write requests are distributed to the corresponding shard server. By directly transmitting data, the cloud platform can actively send commands to the device, ensuring the real-time data upload and command issuance. The TCP access method is built using the Netty framework. Netty is a highly available multiplexed network framework. The bottom layer is implemented using epoll, which can support high concurrent connections and data exchanges, and improve the efficiency and speed of the system. The configuration server stores the routing slice information of the database, and the routing server itself does not store information but reads the cluster configuration from the configuration server into memory at start-up. The slice server is responsible for processing the requests forwarded by the routing server to avoid excessive CPU, memory, and I/O pressure on one server. To ensure data storage security, efficient read and write, and storage system scalability, the system uses a combination of slicing and replica sets, which can ensure the data disaster recovery capability, but also enable the separation of reading and write, while the slicing mode has high scalability, can be extended to cloud computing and other data processing methods, as shown in Figure 5.

As can be seen, by the real-time analysis chart of the cloud platform system, the system can analyze the real-time fluctuation of the data to the size of the data. The data interface also analyses the data in terms of location, and the test device uploads the device coordinates, which need to be converted into the standard coordinates used by the map to improve the accuracy of the display when using Baidu Maps to display the location. The performance of the IoT cloud platform is related to the quality of the services provided by the cloud platform, which needs to be able to run stably under the pressure of high concurrency. The performance of the cloud platform is tested using the JMeter tool, which is an efficient stress testing tool based on Java technology and can simulate huge concurrency to stress test the server. In this article, we first conducted stress tests on the HTTP-based device access interface. In the basic configuration, the connection protocol is HTTP, the system test device access interface listens to port 80, the interface IP address is 192.168.3.232, the interface path is data, the device access interface sends data using POST request to send data, the data are in JSON format, the stress test data include the id of the simulated device, and the simulated data the data include temperature, humidity, latitude, and longitude.

From the test results of the cloud platform, we can see that the maximum response time of the system is 266 ms, the minimum response time is 21 ms, and the average response time is 50 ms. For actual use, due to the equipment conditions and network conditions, the equipment upload cycle is greater than the maximum response time. During the stress test, the system processing error rate is 0.00%, and the request throughput of the system reaches 76.2 times per second, which means that under the operating environment of processing 76.2 requests per second, the average data processing per second reaches 219 KB, and the system is fully capable of correctly processing the data uploaded by the device, as shown in Table 1.

It can be seen that within 100 seconds, the host accepts 4800 TCP requests and the connection is not open; it can be seen that the average processing time of the cloud platform TCP device access server for requests is 54 ms, the minimum processing time is 19 ms, and the longest processing time is 260 ms; for the high real-time requirements of the device, it can fully meet the requirements of the device for real time, and at the same time, the server throughput can reach 47.8 times per second, which means that the server simultaneously processes 47.8 requests per second and 219 bytes of data per second, the error rate of the server is 0.00%, and the system can support normal communication of the usual system in the enterprise.

The platform application module functions, device module main functions, device configuration, issuing commands, and data display module were tested to meet the functional requirements of the cloud platform analysis, and it can fulfill the requirements of equipment access, data processing, data storage, data analysis, and so on. In the performance test, the cloud platform used the JMeter stress test tool to test the data processing capability of the cloud platform in the face of large-scale device access, and the system first used JMeter to test the performance of the HTTP protocol-based device access interface; the average processing time is 50 ms, and the maximum throughput can reach 76.2/sec. Then, the real-time device access interface was tested for performance. The test results show that the processing speed and results of the cloud platform can meet the actual production needs when facing large-scale device access.

### 4.2. Information Mining Precision Marketing Results

In the big data industry chain, the core competencies vary due to the different positions of the alliance members and have the following characteristics: members located in the downstream of the industry chain usually have strong capabilities in data collection, processing, and handling; members located in the midstream of the industry chain usually have strong capabilities in analysis, technology, and model building; and members located in the upstream of the industry chain usually have strong comprehensive capabilities and decision analysis, and knowledge expression capabilities. Therefore, the big data alliance establishes a capacity collaborative scheduling mechanism to give full play to the capacity advantages of each member and promote the alliance can customize data mining services for users with the optimal capacity combination. The cooperative scheduling guarantee among members is an organizational form of scheduling guarantee based on resource collaborative scheduling and capability collaborative scheduling. Through collaborative scheduling among members, it facilitates experience sharing, knowledge exchange, and member interaction to stimulate group wisdom. Therefore, the big data alliance establishes the members' collaborative guarantee mechanism to realize the integration of multiple subjects' wisdom and thus create high value in the process of realizing customized data mining services.

**Figure 6:**
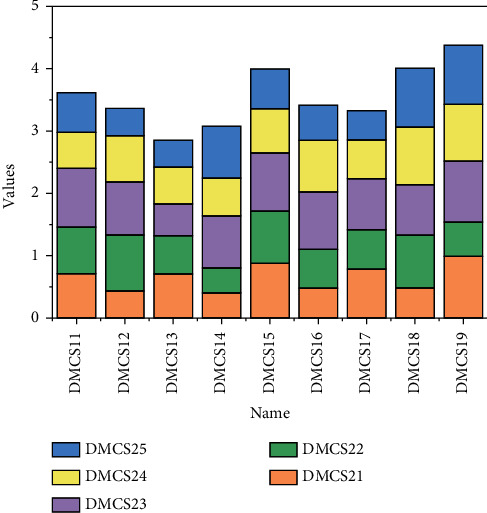
Capability vector distance.

When a user makes a data mining service request, their service request needs to be analyzed and service searched. Retrieve whether there are similar historical service cases in the service case library of the big data alliance data mining service platform. If similar historical service cases exist, then service acquisition needs to be performed in the selected intelligent data mining service model, while if there are no service cases like the target case in the big data alliance historical service case library, then service acquisition needs to be performed using customized data mining services, as shown in Figure 6.

Big data alliance customized data mining services are provided by data resource-based enterprises, data technology-based enterprises, and data application-based enterprises to provide users with more targeted and personalized customized data mining services when the standardized data mining service content cannot meet user needs. The gateway receives the command and encapsulates the command into a command frame and sends it to the device. The device confirms after receiving the command and sends a command confirmation frame to close the connection. The gateway sends the command confirmation message to the cloud platform. The sending order list is transferred to the already issued order list.

**Figure 7:**
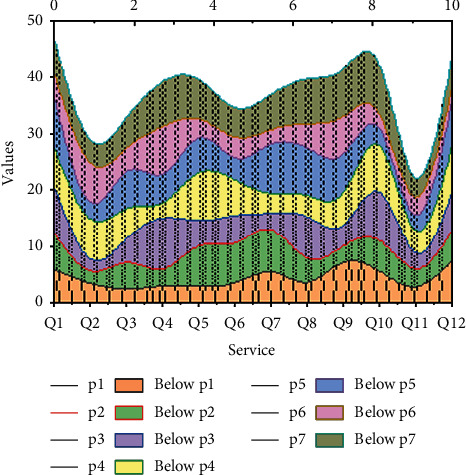
Results of the business model selection problem attributes.

Firstly, the guiding role of user-user needs should be clarified, and the construction of a feedback system for data aggregation services at the decision-making level should be emphasized as the key to the orderly development of the big data alliance. Secondly, the layout of service facilities should be scientifically formulated and reasonably planned, the allocation of service resources should be optimized, efforts should be made to cover a full range of user needs, and multilevel service feedback channels and channels should be developed. Finally, for the same user's service feedback information should pay attention to the collection from multiple channels, to form a closed-loop complete circuit of data aggregation services at the decision-making level, as shown in Figure 7.

As an emerging decision support system, the decision-level data aggregation service model extends the scope of its users to governments, enterprises, organizations, individuals, etc. Especially for enterprises, it not only supports strategic decisions of middle and senior management but also serves front-line business personnel, even including suppliers and partners. The decision-level data aggregation service model assigns service tasks to specific member enterprises through the data aggregation service platform, which requires continuous cooperation and a high degree of collaboration between different types of member enterprises so that the relevant service business content is agreed upon and the consensus is reached among the service subjects without easy ambiguity, and the joint provision of intelligence support products, design support products, selection support products, and implementation support products service solutions.

## 5. Conclusion

For the heterogeneous characteristics of data, the combination of the MongoDB database and MySQL database is used to store data in data storage, which achieves efficient storage and fast access of massive IoT data, and optimizes the data storage method for the temporal nature of IoT data, and stores the data in aggregation to improve the data reading speed. For the storage of massive IoT data and data security, the cloud platform designs a MongoDB database cluster for IoT data storage, which uses a combination of sharing and replica sets to improve the scalability of data storage and the processing capability of a large amount of data storage access. Through the analysis of the big data marketing environment, its advantages in big data marketing are greater than its disadvantages, and with the guidance of government policies and enterprises for their own development needs, big data marketing in Gansu also has a better external environment, to seize market opportunities through differentiated marketing strategies. Based on the problems and shortcomings of the big data marketing strategy, the target market of big data marketing is repositioned, and it is concluded that the government-industry, tourism industry, financial industry, and SME customer market are the key target markets of big data, and for these target markets, the marketing strategy that is more suitable for the long-term development of big data marketing is designed, that is, the strategies of product creation differentiation and operational process differentiation. In this way, we can enhance the comprehensive competitiveness of big data.

## Figures and Tables

**Table 1:** Platform performance.

Label	Sample	Average	Max
HTTP	4.41	3.18	4.75
Request	2.46	6.12	8.82
Total	5.71	8.62	8.47
Mean	3.24	8.94	8.41

## Data Availability

The data used to support the findings of this study are available from the corresponding author upon request.
